# Investigating Electromechanical Buckling Response of FG-GPL-Reinforced Piezoelectric Doubly Curved Shallow Shells Embedded in an Elastic Substrate

**DOI:** 10.3390/ma16082975

**Published:** 2023-04-08

**Authors:** Fatemah H. H. Al Mukahal, Mohammad Alakel Abazid, Mohammed Sobhy

**Affiliations:** 1 Department of Mathematics and Statistics, College of Science, King Faisal University, P.O. Box 400, Al-Ahsa 31982, Saudi Arabia; 2 Department of Mathematics, Faculty of Science, Kafrelsheikh University, Kafrelsheikh 33516, Egypt

**Keywords:** compressive load, four-variable shear deformation shell theory, functionally graded graphene platelets, piezoelectric, doubly curved shallow shells

## Abstract

This work reports the investigations of the electric potential impacts on the mechanical buckling of the piezoelectric nanocomposite doubly curved shallow shells reinforced by functionally gradient graphene platelets (FGGPLs). A four-variable shear deformation shell theory is utilized to describe the components of displacement. The present nanocomposite shells are presumed to be rested on an elastic foundation and subject to electric potential and in-plane compressive loads. These shells are composed of several bonded layers. Each layer is composed of piezoelectric materials strengthened by uniformly distributed GPLs. The Halpin–Tsai model is employed to calculate the Young’s modulus of each layer, whereas Poisson’s ratio, mass density, and piezoelectric coefficients are evaluated based on the mixture rule. The graphene components are graded from one layer to another according to four different piecewise laws. The stability differential equations are deduced based on the principle of virtual work. To test the validity of this work, the current mechanical buckling load is analogized with that available in the literature. Several parametric investigations have been performed to demonstrate the effects of the shell geometry elastic foundation stiffness, GPL volume fraction, and external electric voltage on the mechanical buckling load of the GPLs/piezoelectric nanocomposite doubly curved shallow shells. It is found that the buckling load of GPLs/piezoelectric nanocomposite doubly curved shallow shells without elastic foundations is reduced by increasing the external electric voltage. Moreover, by increasing the elastic foundation stiffness, the shell strength is enhanced, leading to an increase in the critical buckling load.

## 1. Introduction

Symmetric polymeric materials play a key role in a wide range of industrial applications, including chemical assays, the manufacture of microelectronic devices, and petroleum instruments. However, the structure of such materials is comparatively delicate and has restrictive properties that confine their utilization in such sensitive implementations. In order to enhance the exceptional features of such materials, they have been exploited and strengthened with various reinforcements, including nanotubes of boron-nitride [1], piezoelectric and piezoelectromagnetic fibrous materials [2,3], and graphene platelets (GPLs) [4]. The extraordinary kind of composite structure with piezoelectric materials is intensively characterized by electromechanical coupling features along with their effective ability to transform the main energy and production of electrical currents from applied mechanical stresses [5,6,7,8,9]. Accordingly, notable research efforts were carried out to focus on the behavior of such materials, employing functionally graded materials (FGMs) more effectively, as demonstrated by Carl et al. [10]. El Harti et al. [11] used the Euler–Bernoulli theory and finite element method to demonstrate the vibrational control of an FG porous beam with bonded piezoelectric materials in a thermal environment, in which the motion equations were formulated using the Hamilton principle. Working in the context of the first-order shear deformation theory (FSDT), Mallek et al. [12] analyzed a geometrically nonlinear finite shell element to present shells of FG piezolaminated carbon nanotube-reinforced composite (FG-CNTRC). By means of a quasi-3D refined plate theory and the impacts of external electric voltage, Sobhy and Al Mukahal [13] reported the analysis of vibrational behavior of functionally graded piezoelectric plates under 2D magnetic field effects. Garg et al. [14] proposed the bending response of FG sandwich beams subjected to the mechanical load as well as hygrothermal conditions. Furthermore, on the basis of the nonlocal strain gradient (NSGT) and sinusoidal theory, Abazid [5] mainly focused on the buckling and dynamical behavior of a piezoelectromagnetic nanoplate in a hygrothermal medium lying on elastic foundations.

Graphene contains two-dimensional thick layer of carbon atoms bonded together in a hexagonal construction [15]. Besides its low mass density in addition to extraordinary electromechanical properties such as high strength and high Young’s modulus, graphene can be employed as an excellent reinforcement of a variety of matrices, such as polymers and ceramics. Rafiee et al. [16] studied the buckling of a graphene epoxy nanocomposite beam and showed that the obtained buckling load increases gradually with a very small proportion of increase in the weight fraction of GPLs. Sobhy and Abazid [17] demonstrated the effects of the longitudinal magnetic field and external compressions on the vibrational behavior and mechanical buckling analyses of an FGGPL-reinforced sandwich deep curved nanobeam with viscoelastic core with the use of NSGT. Moreover, Abazid [18] elucidated the thermal buckling of metal foam reinforced with FGGPL nanoplates lying on a Pasternak foundation in a humid medium. In particular, the components of displacement were derived by utilizing the modified Reddy’s plate theory and the governing equations were deduced using the NSGT with the help of the principle of virtual displacement. In addition to this, research on graphene has now broadened considerably, utilizing it as an ideal reinforcement of piezoelectric composite structures providing the desirable quality of electromechanical characteristics and stiffness [19,20,21,22]. In the presence of piezoconductive properties, the obtained results of Mao and Zhang [23] illustrated that the GPL nanofillers can remarkably improve the stiffness of the FGGPL-reinforced piezoelectric plates. Furthermore, buckling and post-buckling investigations of FGGPL-reinforced piezoelectric plates subjected to an electric potential and mechanical loads were illustrated by Mao and Zhang [24] in which the equations of motion were solved by the combination of the differential quadrature approach and direct iterative technique. Sobhani and Avcar [25] studied the impact of different carbon-based nano-reinforcements on the frequency analysis of various shells resting on elastic foundations under general boundary conditions. Refs. [6,26,27,28,29] provide a representative selection of other studies on the different behaviors of piezoelectric FGGPLs.

Doubly curved nanocomposite shell panels have been used in considerable various applications in modern industries. Typical examples of shell constructions include automobile bodies, high-pressure vessels, airplane wings, nuclear devices, branching and intersecting pipelines, submarine hulls, etc. However, in order to extend the fundamental equations of the considerable buckling of such shells, some initial investigations are required. For this purpose, it is important to improve the appropriate models for shell manufacturing, especially when they are subjected to dynamic external loads. As a result, notable research efforts have been carried out in the literature to consider the mechanical properties of composite doubly curved shells reinforced by FGGPLs. Duc et al. [30] conducted research on the vibrational behavior of imperfect FGM-thick doubly curved shallow shells integrated with piezoelectric actuators. In this work, the nonlinear dynamic response of such shells resting on an elastic foundation and exposed to various loads containing electro-thermomechanical and damping loadings was studied. According to the analytical method, static and dynamic analyses of FGM doubly curved panels lying on an elastic foundation were provided by Kiani et al. [31]. Moreover, the third-order shear deformation theory (TOSD), without taking into account the thickness stretching effect but retaining all the nonlinear terms in the various variables, has been applied by Amabili [32]. Sobhy [33] studied the magneto-electrothermal bending of composite doubly curved shallow shells integrated with piezoelectromagnetic sheets under several boundary conditions. With the aid of the Ritz approach and Chebyshev polynomials as the main shape functions, Esmaeili and Kiani [34] explored the effects of FGGPL-reinforced doubly curved shells subjected to prompt surface heating. Furthermore, by employing Airy’s stress function and Galerkin’s method, Hoangc et al. [35] elucidated the control effects of the elastic foundation and GPL weight fraction on the nonlinear vibrational behavior of simply supported FG-GPLRC doubly curved shallow shells. Karimiasl et al. [36] demonstrated the comparisons between thermal buckling and post-buckling of FGGPL doubly curved composite shells embedded with shape memory alloy wires using the Halpin–Tsai model. Their analysis was obtained by using TOSD with consideration of von Kármán–Donnell geometric nonlinearity. Recently, Salehipour et al. [37] investigated the stability of doubly curved shells resting on an elastic foundation and made of laminated composites reinforced by carbon fibers, CNTs, and GPLs based on the Galerkin method.

As shown in the previous investigations, no paper has studied piezoelectric nanocomposite doubly curved shallow shells reinforced by FGGPLs. In addition, piezoelectric materials have been employed in a wide range of fields such as actuators, sensors, medicine, the aerospace industry, smart devices, and micro-electromechanical systems. For producing powerful piezoelectric devices without any bonding agent, the piezoelectric materials are strengthened by GPLs. Motivated by the need for greater understanding of such nanocomposite shells, the current research attempts to analyze the electric potential effects on the mechanical buckling of the piezoelectric nanocomposite doubly curved shallow shells reinforced by FGGPLs. By employing a four-variable shear deformation shell theory, the displacement field is described. The present nanocomposite shells are presumed to be rested on an elastic substrate and exposed to electric potential in addition to the in-plane compressive loads. The present shell is composed of various bonded composite layers in which each layer is composed of piezoelectric materials strengthened by uniformly distributed GPLs. The Young’s modulus of each layer is calculated based upon the Halpin–Tsai model, whereas Poisson’s ratio, mass density, and piezoelectric coefficients are estimated based on the mixture rule. Furthermore, according to four different piecewise laws, the graphene components are graded from one layer to another. The stability differential equations are obtained by using the principle of virtual work. To test the validity of the mechanical buckling load, the current results are compared closely with those available in the literature. Furthermore, various parametric studies are discussed to demonstrate the impact of the shell geometry, elastic foundation stiffness, GPL volume fraction, and external electric voltage on the mechanical buckling load of the GPLs/piezoelectric nanocomposite doubly curved shallow shells.

## 2. Problem Formulation

### 2.1. Shell Configuration

**Figure 1:**
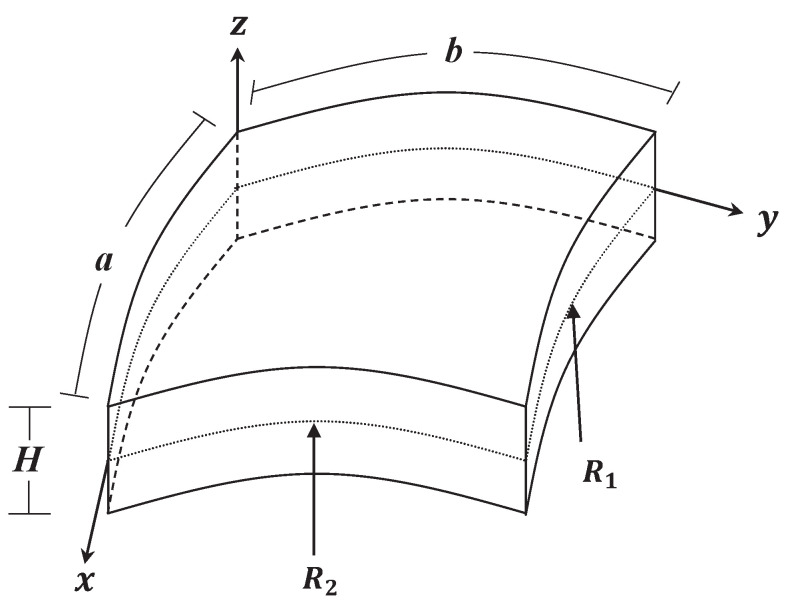
Geometry and coordinates of a doubly curved shallow shell.

**Figure 2:**
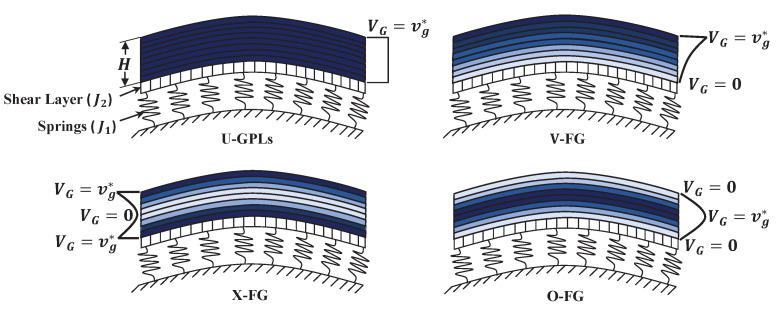
Doubly curved shallow shells with different GPL distributions resting on elastic foundations.

We consider in this research a doubly curved shallow shell composed of multiple nanocomposite homogeneous layers that have the length *a*, width *b*, and total thickness *H*, as shown in Figure 1. Each layer is a mixture of a piezoelectric polymer as the matrix and GPLs as a reinforcement. The GPLs are uniformly spread throughout every layer, whereas the volume fraction varies from one layer to another. The deformations of the doubly curved shell are described by the coordinates (x,y,z). The middle surface of the shell coincides with z=0. The radii of the principal curvature of the middle surface of the panel are indicated by $R_{1}$ and $R_{2}$. With regard to a modified law of distribution of GPLs throughout the thickness of the panel, four distinct types (see Figure 2) are provided as follows:

(1) $$\begin{array}{c} V_{G}^{\left(i\right)}=\left\{\begin{array}{ccc} v_{g}^{*}, & & \mathrm{for}\;\mathrm{U-GPLs};\\ v_{g}^{*}{\left(\frac{i-1}{K-1}\right)}^{n}, & \forall K\in N,K>1, & \mathrm{for}\;\mathrm{V-FG};\\ v_{g}^{*}{\left(\frac{|2i-K-1|-1}{K-2}\right)}^{n}, & \forall K\in N_{\mathrm{even}},K>2\;, & \mathrm{for}\;\mathrm{X-FG};\\ v_{g}^{*}{\left(\frac{|2i-K-1|+1-K}{2-K}\right)}^{n}, & \forall K\in N_{\mathrm{even}},K>2\;, & \mathrm{for}\;\mathrm{O-FG}, \end{array}\right. \end{array}$$

where *K* represents the number of layers, *n* is the power law index, and $v_{g}^{*}$ is defined as [38,39]

(2) $$\begin{array}{c} v_{g}^{*}=\frac{\rho_{m}w_{f}}{\rho_{m}w_{f}+\rho_{G}\left(1-w_{f}\right)}, \end{array}$$

in which $w_{f}$ stands for the weight fraction of the GPLs; $\rho_{m}$ and $\rho_{G}$ represent the densities of the matrix and GPLs, respectively. The modified Halpin–Tsai model [40] is implemented so that the effective Young’s modulus of the shells is calculated as:

(3) $$\begin{array}{c} \begin{array}{cc} \; & E^{\left(i\right)}=\frac{3}{8}\Gamma_{1}E^{m}+\frac{5}{8}\Gamma_{2}E^{m},\\ \; & \Gamma_{r}=\frac{1+2A_{r}\eta_{r}V_{G}^{\left(i\right)}}{1-\eta_{r}V_{G}^{\left(i\right)}},\\ \; & \eta_{r}=\frac{E^{G}-E^{m}}{E^{G}+2E^{m}A_{r}},\;r=1,2, \end{array} \end{array}$$

where $A_{1}=a_{G}/h_{G}$ and $A_{2}=b_{G}/h_{G}$, in which $a_{G},\;b_{G}$, and $h_{G}$ stand for the length, width, and thickness of the GPLs, respectively; $E^{G}$ and $E^{m}$ are Young’s moduli of the GPLs and matrix, respectively. However, Poisson’s ratio $\nu^{\left(i\right)}$, the piezoelectric coefficient $e_{kl}$, and the dielectric coefficient $g_{kk}$ are given by

(4) $$\begin{array}{c} \begin{array}{cc} \; & \nu^{\left(i\right)}=V_{G}^{\left(i\right)}\nu_{G}+\left(1-V_{G}^{\left(i\right)}\right)\nu_{m},\\ \; & e_{kl}^{\left(i\right)}=V_{G}^{\left(i\right)}e_{kl}^{G}+\left(1-V_{G}^{\left(i\right)}\right)e_{kl}^{m},\;k,l=1,2,3,4,5,\\ \; & g_{kk}^{\left(i\right)}=V_{G}^{\left(i\right)}g_{kk}^{G}+\left(1-V_{G}^{\left(i\right)}\right)g_{kk}^{m},\;k=1,2,3. \end{array} \end{array}$$

where $\nu_{G}$ and $\nu_{m}$ represent the Poisson’s ratios of the GPLs and matrix, $e_{kl}^{G}$ and $e_{kl}^{m}$ are the piezoelectric coefficients of the GPLs and matrix, $g_{kk}^{G}$ and $g_{kk}^{m}$ are the dielectric coefficients of the GPLs and matrix, respectively.

### 2.2. Displacement Field

The displacement components are given by [41]

(5) $$\begin{array}{cc} \; & U\left(x,y,z\right)=\left(1+\frac{z}{R_{1}}\right)\;u_{0}\left(x,y\right)-z\;\frac{\partial w_{1}}{\partial x}-\phi \left(z\right)\frac{\partial w_{2}}{\partial x},\\ \; & V\left(x,y,z\right)=\left(1+\frac{z}{R_{2}}\right)\;v_{0}\left(x,y\right)-z\;\frac{\partial w_{1}}{\partial y}-\phi \left(z\right)\frac{\partial w_{2}}{\partial y},\\ \; & W\left(x,z\right)=w_{1}\left(x,y\right)+w_{2}\left(x,y\right),\\ \; & \phi (z)=z-f(z) \end{array}$$

where $u_{0}$ and $v_{0}$ denote the mid-plane displacement components in the directions of x- and y-axes, respectively. The transverse deflection *W* is divided into two components $w_{1}$ and $w_{2}$ that indicate the bending and shear displacements, respectively. Furthermore, the function f(z) indicates the configuration of the shear stress throughout the thickness of the shallow shells, which can be given as follows:

(6) $$f\left(z\right)=\frac{z}{1+{\left(z/H\right)}^{2}}-\frac{5}{8}\frac{z^{3}}{H^{2}}.$$

In accordance with the shape function (6), the transverse shear stress can take a parabolic form throughout the thickness of the panel. Thus, the shear correction factors are no longer required, since the correct representation of the transverse shear strain is given. Furthermore, it can satisfy the traction-free boundary conditions at the panel faces. Moreover, the above shape function (6) foresees precise outcomes as discussed in [42,43,44].

By considering the displacement field (5), the non-zero components of the strains are obtained as [45,46]:

(7) $$\begin{array}{cc} \varepsilon_{11} & =\gamma_{11}^{\left(0\right)}+z\gamma_{11}^{\left(1\right)}+\phi \gamma_{11}^{\left(2\right)},\\ \varepsilon_{22} & =\gamma_{22}^{\left(0\right)}+z\gamma_{22}^{\left(1\right)}+\phi \gamma_{22}^{\left(2\right)},\\ \varepsilon_{12} & =\gamma_{12}^{\left(0\right)}+z\gamma_{12}^{\left(1\right)}+\phi \gamma_{12}^{\left(2\right)},\\ \varepsilon_{23} & =f^{{}^{\prime }}\left(z\right)\gamma_{23}^{\left(2\right)},\\ \varepsilon_{13} & =f^{{}^{\prime }}\left(z\right)\gamma_{13}^{\left(2\right)},\;f^{{}^{\prime }}\left(z\right)=\frac{df}{dz}, \end{array}$$

where

(8) $$\begin{array}{cc} \gamma_{11}^{\left(0\right)} & =\frac{\partial u_{0}}{\partial x}+\frac{w_{1}+w_{2}}{R_{1}},\;\gamma_{22}^{\left(0\right)}=\frac{\partial v_{0}}{\partial y}+\frac{w_{1}+w_{2}}{R_{2}},\;\gamma_{12}^{\left(0\right)}=\frac{\partial v_{0}}{\partial x}+\frac{\partial u_{0}}{\partial y},\\ \gamma_{11}^{\left(1\right)} & =-\frac{\partial^{2}w_{1}}{\partial x^{2}},\;\gamma_{22}^{\left(1\right)}=-\frac{\partial^{2}w_{1}}{\partial y^{2}},\;\gamma_{12}^{\left(1\right)}=-2\frac{\partial^{2}w_{1}}{\partial x\partial y},\\ \gamma_{11}^{\left(2\right)} & =-\frac{\partial^{2}w_{2}}{\partial x^{2}},\;\gamma_{22}^{\left(2\right)}=-\frac{\partial^{2}w_{2}}{\partial y^{2}},\;\gamma_{12}^{\left(2\right)}=-2\frac{\partial^{2}w_{2}}{\partial x\partial y},\\ \gamma_{13}^{\left(2\right)} & =\frac{\partial w_{2}}{\partial x},\;\gamma_{23}^{\left(2\right)}=\frac{\partial w_{2}}{\partial y}. \end{array}$$

### 2.3. Constitutive Relations

Based on the piezoelasticity theory [6,47], the component constitutive relations of the stresses σ can be given by

(9) $${\left\{\begin{array}{c} \sigma_{1}\\ \sigma_{2}\\ \sigma_{4}\\ \sigma_{5}\\ \sigma_{6} \end{array}\right\}}^{\left(i\right)}={\left[\begin{array}{ccccc} {\tilde{E}}_{11} & {\tilde{E}}_{12} & 0 & 0 & 0\\ {\tilde{E}}_{12} & {\tilde{E}}_{22} & 0 & 0 & 0\\ 0 & 0 & {\tilde{E}}_{44} & 0 & 0\\ 0 & 0 & 0 & {\tilde{E}}_{55} & 0\\ 0 & 0 & 0 & 0 & {\tilde{E}}_{66} \end{array}\right]}^{\left(i\right)}\left\{\begin{array}{c} \varepsilon_{11}\\ \varepsilon_{22}\\ \varepsilon_{23}\\ \varepsilon_{13}\\ \varepsilon_{12} \end{array}\right\}-{\left[\begin{array}{ccc} 0 & 0 & e_{31}\\ 0 & 0 & e_{32}\\ 0 & e_{24} & 0\\ e_{15} & 0 & 0\\ 0 & 0 & 0 \end{array}\right]}^{\left(i\right)}{\left\{\begin{array}{c} E_{x}\\ E_{y}\\ E_{z} \end{array}\right\}}^{\left(i\right)},$$

where the elastic coefficients of the FGGPL nanocomposite layers of the doubly curved shallow shell are written as

(10) $$\begin{array}{c} {\tilde{E}}_{11}^{\left(i\right)}={\tilde{E}}_{22}^{\left(i\right)}=\frac{E^{\left(i\right)}}{1-{\left(\nu^{\left(i\right)}\right)}^{2}},\;{\tilde{E}}_{12}^{\left(i\right)}=\frac{\nu^{\left(i\right)}E^{\left(i\right)}}{1-{\left(\nu^{\left(i\right)}\right)}^{2}},\;{\tilde{E}}_{44}^{\left(i\right)}={\tilde{E}}_{55}^{\left(i\right)}={\tilde{E}}_{66}^{\left(i\right)}=\frac{E^{\left(i\right)}}{2\left(1+\nu^{\left(i\right)}\right)}. \end{array}$$

The electric displacement $D_{i}$ can be given as [6]

(11) $${\left\{\begin{array}{c} D_{x}\\ D_{y}\\ D_{z} \end{array}\right\}}^{\left(i\right)}={\left[\begin{array}{ccccc} 0 & 0 & 0 & e_{15} & 0\\ 0 & 0 & e_{24} & 0 & 0\\ e_{31} & e_{32} & 0 & 0 & 0 \end{array}\right]}^{\left(i\right)}\left\{\begin{array}{c} \varepsilon_{11}\\ \varepsilon_{22}\\ \varepsilon_{23}\\ \varepsilon_{13}\\ \varepsilon_{12} \end{array}\right\}+{\left[\begin{array}{ccc} g_{11} & 0 & 0\\ 0 & g_{22} & 0\\ 0 & 0 & g_{33} \end{array}\right]}^{\left(i\right)}{\left\{\begin{array}{c} E_{x}\\ E_{y}\\ E_{z} \end{array}\right\}}^{\left(i\right)}.$$

The electric field E can be given as

(12) E=−∇Ψ,

where Ψ represents the electric potential in which it is supposed to be as a combination of linear and trigonometric variations; in particular, it can be defined as [6]

(13) $$\Psi \left(x,y,z\right)=\frac{2z}{H}\Psi_{0}-\bar{\Psi }\left(x,y\right)\cos\left(\frac{\pi z}{H}\right),$$

in which $\Psi_{0}$ is the external applied voltage and $\bar{\Psi }$ is the electric potential. Substituting Equation (12) into Equation (13) gives us the electric field as follows:

(14) $$\begin{array}{cc} E_{x} & =\frac{\partial \bar{\Psi }}{\partial x}\cos\left(\frac{\pi z}{H}\right),\\ E_{y} & =\frac{\partial \bar{\Psi }}{\partial y}\cos\left(\frac{\pi z}{H}\right),\\ E_{z} & =-\frac{2\Psi_{0}}{H}-\frac{\pi }{H}\bar{\Psi }\left(x,y\right)\sin\left(\frac{\pi z}{H}\right). \end{array}$$

## 3. Governing Equations

The governing equations are deduced by utilizing the virtual work principle that can be expressed as

(15) $$\begin{array}{c} \delta \Pi_{\mathrm{SE}}-\delta \Pi_{\mathrm{FE}}=0, \end{array}$$

where the variation of the strain energy $\delta \Pi_{\mathrm{SE}}$ and the work done by the external force $\delta \Pi_{\mathrm{FE}}$ are given as

(16) $$\begin{array}{cc} \; & \delta \Pi_{\mathrm{SE}}=\sum_{j=1}^{K}\int_{{\tilde{h}}_{j}}^{{\tilde{h}}_{j+1}}\int_{A}(\sigma_{1}^{\left(j\right)}\delta \varepsilon_{11}+\sigma_{2}^{\left(j\right)}\delta \varepsilon_{22}+\sigma_{12}^{\left(j\right)}\delta \varepsilon_{12}+\sigma_{23}^{\left(j\right)}\delta \varepsilon_{23}+\sigma_{13}^{\left(j\right)}\delta \varepsilon_{13}\\ \; & \;\;-D_{x}^{\left(j\right)}\delta E_{x}-D_{y}^{\left(j\right)}\delta E_{y}-D_{z}^{\left(j\right)}\delta E_{z})dAdz,\\ \; & \delta \Pi_{\mathrm{FE}}=\int_{A}\left[F_{x}\frac{\partial^{2}\left(w_{1}+w_{2}\right)}{\partial x^{2}}+F_{y}\frac{\partial^{2}\left(w_{1}+w_{2}\right)}{\partial y^{2}}+N_{1}^{0}\nabla^{2}\left(w_{1}+w_{2}\right)\right]\delta \left(w_{1}+w_{2}\right)dA\\ \; & \;\;-\int_{A}\left[J_{1}\left(w_{1}+w_{2}\right)-J_{2}\nabla^{2}\left(w_{1}+w_{2}\right)\right]\delta \left(w_{1}+w_{2}\right)dA,\\ \; & {\tilde{h}}_{j}=-\left(\frac{K}{2}-j+1\right)\frac{H}{K},\;j=1,2,......,K+1, \end{array}$$

where $F_{x}$ and $F_{y}$ stand for the in-plane compressive loads applied along the *x* and *y* axes, $J_{1}$ and $J_{2}$ are the elastic foundation coefficients, and $N_{1}^{0}$ is the in-plane electric force that can be given as

(17) $$\begin{array}{c} N_{1}^{0}=\sum_{j=1}^{K}\int_{{\tilde{h}}_{j}}^{{\tilde{h}}_{j+1}}e_{31}^{\left(j\right)}\frac{2\Psi_{0}}{H}dz, \end{array}$$

where ${\tilde{h}}_{j}$ and ${\tilde{h}}_{j+1}$ denote the interface coordinates between the layers *j* and j+1. By substituting Equation (7) into Equation (16), one obtains the variation of the strain energy as

(18) $$\begin{array}{cc} \delta \Pi_{\mathrm{SE}}= & \int_{A}(N_{1}\delta \gamma_{11}^{\left(0\right)}+M_{1}\delta \gamma_{11}^{\left(1\right)}+{\hat{R}}_{1}\delta \gamma_{11}^{\left(2\right)}+N_{2}\delta \gamma_{22}^{\left(0\right)}+M_{2}\delta \gamma_{22}^{\left(1\right)}+{\hat{R}}_{2}\delta \gamma_{22}^{\left(2\right)}+N_{6}\delta \gamma_{12}^{\left(0\right)}\\ + & M_{6}\delta \gamma_{12}^{\left(1\right)}+{\hat{R}}_{6}\delta \gamma_{12}^{\left(2\right)}+Q_{5}\delta \gamma_{13}^{\left(2\right)}+Q_{4}\delta \gamma_{23}^{\left(2\right)}-S_{1}\delta \frac{\partial \bar{\Psi }}{\partial x}-S_{2}\delta \frac{\partial \bar{\Psi }}{\partial y}+S_{3}\delta \bar{\Psi })dA, \end{array}$$

where

(19) $$\begin{array}{cc} \{N_{l},M_{l},{\hat{R}}_{l}\}= & \sum_{j=1}^{K}\int_{{\tilde{h}}_{j}}^{{\tilde{h}}_{j+1}}\sigma_{l}^{\left(j\right)}\left\{1,z,\phi \right\}dz,\;l=1,2,6\\ Q_{\alpha }= & \sum_{j=1}^{K}\int_{{\tilde{h}}_{j}}^{{\tilde{h}}_{j+1}}\sigma_{\alpha }^{\left(j\right)}f^{\prime }dz,\;\alpha =4,5\\ S_{1}= & \sum_{j=1}^{K}\int_{{\tilde{h}}_{j}}^{{\tilde{h}}_{j+1}}D_{x}^{\left(j\right)}\cos\left(\frac{\pi z}{H}\right)dz,\\ S_{2}= & \sum_{j=1}^{K}\int_{{\tilde{h}}_{j}}^{{\tilde{h}}_{j+1}}D_{y}^{\left(j\right)}\cos\left(\frac{\pi z}{H}\right)dz,\\ S_{3}= & \sum_{j=1}^{K}\int_{{\tilde{h}}_{i}}^{{\tilde{h}}_{j+1}}D_{z}^{\left(j\right)}\frac{\pi }{H}\sin\left(\frac{\pi z}{H}\right)dz. \end{array}$$

The governing equations of piezoelectric nanocomposite doubly curved shallow shells are attained neighboring the equilibrium state. For this purpose, the displacement components are supposed to have two components [48,49,50]:

(20) $$\begin{array}{c} u_{0}=u_{0}^{0}+u_{0}^{1},\;v_{0}=v_{0}^{0}+v_{0}^{1},\;w_{1}=w_{1}^{0}+w_{1}^{1},\;w_{2}=w_{2}^{0}+w_{2}^{1}, \end{array}$$

where ($u_{0}^{0},v_{0}^{0},w_{1}^{0},w_{2}^{0}$) are the equilibrium state displacements, while ($u_{0}^{1},v_{0}^{1},w_{1}^{1},w_{2}^{1}$) stand for the virtual displacements of a neighboring stable state. In addition, the corresponding resultants of the stress and couple stress can be expressed as

(21) $$\begin{array}{cc} \; & N_{l}=N_{l}^{0}+N_{l}^{1},\;M_{l}=M_{l}^{0}+M_{l}^{1},\;{\hat{R}}_{l}={\hat{R}}_{l}^{0}+{\hat{R}}_{l}^{1},\;l=1,2,6,\\ \; & Q_{\alpha }=Q_{\alpha }^{0}+Q_{\alpha }^{1},\;S_{r}=S_{r}^{0}+S_{r}^{1},\;\alpha =4,5,\;r=1,2,3. \end{array}$$

Substituting Equations (16) and (18) into Equation (15) subject to Equations (20) and (21) yields the governing equations:

(22) $$\begin{array}{cc} \; & \frac{\partial N_{1}^{1}}{\partial x}+\frac{\partial N_{6}^{1}}{\partial y}=0,\\ \; & \frac{\partial N_{6}^{1}}{\partial x}+\frac{\partial N_{2}^{1}}{\partial y}=0,\\ \; & \frac{\partial^{2}M_{1}^{1}}{\partial x^{2}}+2\frac{\partial^{2}M_{6}^{1}}{\partial x\partial y}+\frac{\partial^{2}M_{2}^{1}}{\partial y^{2}}-\frac{N_{1}^{1}}{R_{1}}-\frac{N_{2}^{1}}{R_{2}}+F_{x}\frac{\partial^{2}\left(w_{1}^{1}+w_{2}^{1}\right)}{\partial x^{2}}+F_{y}\frac{\partial^{2}\left(w_{1}^{1}+w_{2}^{1}\right)}{\partial y^{2}}\\ \; & \;+N_{1}^{0}\nabla^{2}\left(w_{1}^{1}+w_{2}^{1}\right)-J_{1}\left(w_{1}^{1}+w_{2}^{1}\right)+J_{2}\nabla^{2}\left(w_{1}^{1}+w_{2}^{1}\right)=0,\\ \; & \frac{\partial^{2}{\hat{R}}_{1}^{1}}{\partial x^{2}}+2\frac{\partial^{2}{\hat{R}}_{6}^{1}}{\partial x\partial y}+\frac{\partial^{2}{\hat{R}}_{2}^{1}}{\partial y^{2}}+\frac{\partial Q_{5}^{1}}{\partial x}+\frac{\partial Q_{4}^{1}}{\partial y}-\frac{N_{1}^{1}}{R_{1}}-\frac{N_{2}^{1}}{R_{2}}+F_{x}\frac{\partial^{2}\left(w_{1}^{1}+w_{2}^{1}\right)}{\partial x^{2}}\\ \; & \;+F_{y}\frac{\partial^{2}\left(w_{1}^{1}+w_{2}^{1}\right)}{\partial y^{2}}+N_{1}^{0}\nabla^{2}\left(w_{1}^{1}+w_{2}^{1}\right)-J_{1}\left(w_{1}^{1}+w_{2}^{1}\right)+J_{2}\nabla^{2}\left(w_{1}^{1}+w_{2}^{1}\right)=0,\\ \; & \frac{\partial S_{1}^{1}}{\partial x}+\frac{\partial S_{2}^{1}}{\partial y}+S_{3}^{1}=0. \end{array}$$

For convenience, superscript 1 may be neglected.

By inserting Equations (9) and (11) into Equation (19), we obtain the stress resultants as

(23) $$\begin{array}{cc} N_{1}= & N_{11}\gamma_{11}^{\left(0\right)}+N_{12}\gamma_{11}^{\left(1\right)}+N_{13}\gamma_{11}^{\left(2\right)}+{\bar{N}}_{11}\gamma_{22}^{\left(0\right)}+{\bar{N}}_{12}\gamma_{22}^{\left(1\right)}+{\bar{N}}_{13}\gamma_{22}^{\left(2\right)}+B_{11}\bar{\Psi },\\ N_{2}= & {\bar{N}}_{11}\gamma_{11}^{\left(0\right)}+{\bar{N}}_{12}\gamma_{11}^{\left(1\right)}+{\bar{N}}_{13}\gamma_{11}^{\left(2\right)}+N_{11}\gamma_{22}^{\left(0\right)}+N_{12}\gamma_{22}^{\left(1\right)}+N_{13}\gamma_{22}^{\left(2\right)}+B_{11}\bar{\Psi },\\ N_{6}= & {\hat{N}}_{11}\gamma_{12}^{\left(0\right)}+{\hat{N}}_{12}\gamma_{12}^{\left(1\right)}+{\hat{N}}_{13}\gamma_{12}^{\left(2\right)}, \end{array}$$

(24) $$\begin{array}{cc} M_{1}= & N_{12}\gamma_{11}^{\left(0\right)}+N_{22}\gamma_{11}^{\left(1\right)}+N_{23}\gamma_{11}^{\left(2\right)}+{\bar{N}}_{12}\gamma_{22}^{\left(0\right)}+{\bar{N}}_{22}\gamma_{22}^{\left(1\right)}+{\bar{N}}_{23}\gamma_{22}^{\left(2\right)}+B_{12}\bar{\Psi },\\ M_{2}= & {\bar{N}}_{12}\gamma_{11}^{\left(0\right)}+{\bar{N}}_{22}\gamma_{11}^{\left(1\right)}+{\bar{N}}_{23}\gamma_{11}^{\left(2\right)}+N_{12}\gamma_{22}^{\left(0\right)}+N_{22}\gamma_{22}^{\left(1\right)}+N_{23}\gamma_{22}^{\left(2\right)}+B_{12}\bar{\Psi },\\ M_{6}= & {\hat{N}}_{12}\gamma_{12}^{\left(0\right)}+{\hat{N}}_{22}\gamma_{12}^{\left(1\right)}+{\hat{N}}_{23}\gamma_{12}^{\left(2\right)}, \end{array}$$

(25) $$\begin{array}{cc} {\hat{R}}_{1}= & N_{13}\gamma_{11}^{\left(0\right)}+N_{23}\gamma_{11}^{\left(1\right)}+N_{33}\gamma_{11}^{\left(2\right)}+{\bar{N}}_{13}\gamma_{22}^{\left(0\right)}+{\bar{N}}_{23}\gamma_{22}^{\left(1\right)}+{\bar{N}}_{33}\gamma_{22}^{\left(2\right)}+B_{13}\bar{\Psi },\\ {\hat{R}}_{2}= & {\bar{N}}_{13}\gamma_{11}^{\left(0\right)}+{\bar{N}}_{23}\gamma_{11}^{\left(1\right)}+{\bar{N}}_{33}\gamma_{11}^{\left(2\right)}+N_{13}\gamma_{22}^{\left(0\right)}+N_{23}\gamma_{22}^{\left(1\right)}+N_{33}\gamma_{22}^{\left(2\right)}+B_{13}\bar{\Psi },\\ {\hat{R}}_{6}= & {\hat{N}}_{13}\gamma_{12}^{\left(0\right)}+{\hat{N}}_{23}\gamma_{12}^{\left(1\right)}+{\hat{N}}_{33}\gamma_{12}^{\left(2\right)}, \end{array}$$

(26) $$\begin{array}{cc} Q_{4}= & Q_{44}\gamma_{23}^{\left(2\right)}-B_{21}\frac{\partial \bar{\Psi }}{\partial y},\\ Q_{5}= & Q_{44}\gamma_{13}^{\left(2\right)}-B_{21}\frac{\partial \bar{\Psi }}{\partial x},\\ S_{1}= & B_{21}\gamma_{13}^{\left(2\right)}+B_{31}\frac{\partial \bar{\Psi }}{\partial x},\\ S_{2}= & B_{21}\gamma_{23}^{\left(2\right)}+B_{31}\frac{\partial \bar{\Psi }}{\partial y},\\ S_{3}= & B_{11}\left(\gamma_{11}^{\left(0\right)}+\gamma_{22}^{\left(0\right)}\right)+B_{12}\left(\gamma_{11}^{\left(1\right)}+\gamma_{22}^{\left(1\right)}\right)+B_{13}\left(\gamma_{11}^{\left(2\right)}+\gamma_{22}^{\left(2\right)}\right)-B_{33}\bar{\Psi }, \end{array}$$

where

(27) $$\begin{array}{cc} \; & \left\{N_{11},N_{12},N_{13}\right\}=\sum_{j=1}^{K}\int_{{\tilde{h}}_{j}}^{{\tilde{h}}_{j+1}}{\tilde{E}}_{11}^{\left(j\right)}\left\{1,z,\phi \right\}dz,\\ \; & \left\{{\bar{N}}_{11},{\bar{N}}_{12},{\bar{N}}_{13}\right\}=\sum_{j=1}^{K}\int_{{\tilde{h}}_{j}}^{{\tilde{h}}_{j+1}}{\tilde{E}}_{12}^{\left(j\right)}\left\{1,z,\phi \right\}dz,\\ \; & \left\{{\hat{N}}_{11},{\hat{N}}_{12},{\hat{N}}_{13}\right\}=\sum_{j=1}^{K}\int_{{\tilde{h}}_{j}}^{{\tilde{h}}_{j+1}}{\tilde{E}}_{66}^{\left(j\right)}\left\{1,z,\phi \right\}dz,\\ \; & \left\{N_{22},N_{23}\right\}=\sum_{j=1}^{K}\int_{{\tilde{h}}_{j}}^{{\tilde{h}}_{j+1}}{\tilde{E}}_{11}^{\left(j\right)}\left\{z^{2},z\phi \right\}dz,\\ \; & \left\{{\bar{N}}_{22},{\bar{N}}_{23}\right\}=\sum_{j=1}^{K}\int_{{\tilde{h}}_{j}}^{{\tilde{h}}_{j+1}}{\tilde{E}}_{12}^{\left(j\right)}\left\{z^{2},z\phi \right\}dz,\\ \; & \left\{{\hat{N}}_{22},{\hat{N}}_{23}\right\}=\sum_{j=1}^{K}\int_{{\tilde{h}}_{j}}^{{\tilde{h}}_{j+1}}{\tilde{E}}_{66}^{\left(j\right)}\left\{z^{2},z\phi \right\}dz,\\ \; & N_{33}=\sum_{j=1}^{K}\int_{{\tilde{h}}_{j}}^{{\tilde{h}}_{j+1}}{\tilde{E}}_{11}^{\left(j\right)}\phi^{2}dz,\\ \; & {\bar{N}}_{33}=\sum_{j=1}^{K}\int_{{\tilde{h}}_{j}}^{{\tilde{h}}_{j+1}}{\tilde{E}}_{12}^{\left(j\right)}\phi^{2}dz,\\ \; & {\hat{N}}_{33}=\sum_{j=1}^{K}\int_{{\tilde{h}}_{j}}^{{\tilde{h}}_{j+1}}{\tilde{E}}_{66}^{\left(j\right)}\phi^{2}dz,\\ \; & Q_{44}=\sum_{j=1}^{K}\int_{{\tilde{h}}_{j}}^{{\tilde{h}}_{j+1}}{f^{{}^{\prime }}}^{2}\left(z\right){\tilde{E}}_{44}^{\left(j\right)}dz,\\ \; & \left\{B_{11},B_{12},B_{13}\right\}=\sum_{j=1}^{K}\int_{{\tilde{h}}_{j}}^{{\tilde{h}}_{j+1}}e_{31}^{\left(j\right)}\frac{\pi }{H}\left\{1,z,\phi \right\}\sin\left(\frac{\pi z}{H}\right)dz, \end{array}$$

(28) $$\begin{array}{cc} B_{21}= & \sum_{j=1}^{K}\int_{{\tilde{h}}_{j}}^{{\tilde{h}}_{j+1}}f^{{}^{\prime }}\left(z\right)e_{24}^{\left(j\right)}\cos\left(\frac{\pi z}{H}\right)dz,\\ B_{31}= & \sum_{j=1}^{K}\int_{{\tilde{h}}_{j}}^{{\tilde{h}}_{j+1}}g_{11}^{\left(j\right)}\cos^{2}\left(\frac{\pi z}{H}\right)dz,\\ B_{33}= & \sum_{j=1}^{K}\int_{{\tilde{h}}_{j}}^{{\tilde{h}}_{j+1}}g_{33}^{\left(j\right)}\frac{\pi^{2}}{H^{2}}\sin^{2}\left(\frac{\pi z}{H}\right)dz. \end{array}$$

## 4. Solution Procedure

Now the governing equations (22) are solved analytically to get the critical buckling load of the smart nanocomposite doubly curved shallow shells embedded in an elastic substrate. For this purpose, the simply supported boundary conditions along *x* and *y* directions are defined as

(29) $$\begin{array}{c} \begin{array}{cc} \; & v_{0}=w_{1}=w_{2}=\bar{\Psi }=\frac{\partial w_{1}}{\partial y}=\frac{\partial w_{2}}{\partial y}=N_{1}=M_{1}={\hat{R}}_{1}=0,\;\mathrm{at}\;x=0,a,\\ \; & u_{0}=w_{1}=w_{2}=\bar{\Psi }=\frac{\partial w_{1}}{\partial x}=\frac{\partial w_{2}}{\partial x}=N_{2}=M_{2}={\hat{R}}_{2}=0,\;\mathrm{at}\;y=0,b. \end{array} \end{array}$$

The approximate solutions are presented to satisfy the above boundary conditions as follows:

(30) $$\begin{array}{cc} \; & u_{0}=\sum_{k=1}^{\infty }\sum_{s=1}^{\infty }U_{ks}\cos\left(\lambda x\right)\sin\left(\mu y\right),\\ \; & v_{0}=\sum_{k=1}^{\infty }\sum_{s=1}^{\infty }V_{ks}\sin\left(\lambda x\right)\cos\left(\mu y\right),\\ \; & \left\{w_{1},\;w_{2},\;\bar{\Psi }\right\}=\sum_{k=1}^{\infty }\sum_{s=1}^{\infty }\left\{W_{1ks},\;W_{2ks},\;G_{ks}\right\}\sin\left(\lambda x\right)\sin\left(\mu y\right), \end{array}$$

where $U_{ks}$, $V_{ks}$, $W_{1ks}$, $W_{2ks}$, and $G_{ks}$ are unknown functions, λ=kπ/a, μ=sπ/b, where *k* and *s* are the mode numbers. For the present analysis, $F_{x}=-\hat{F}$ and $F_{y}=-\zeta \hat{F}$. Substituting Equation (30) into Equation (22) gives the following eigenvalue problem:

(31) $$\begin{array}{cc} \; & \left[\begin{array}{c} L \end{array}\right]\left\{\begin{array}{c} \Lambda \end{array}\right\}=0, \end{array}$$

where

(32) $$\begin{array}{c} \Lambda ={\left\{U_{ks},\;V_{ks},\;W_{1ks},\;W_{2ks},\;G_{ks}\right\}}^{T}, \end{array}$$

and the entries $L_{ij}$ of the matrix [L] are represented by

(33) $$\begin{array}{cc} \; & L_{11}=-N_{11}\lambda^{2}-{\hat{N}}_{11}\mu^{2},\;L_{12}=-\left({\bar{N}}_{11}+{\hat{N}}_{11}\right)\lambda \mu =L_{21},\\ \; & L_{13}=N_{12}\lambda^{3}+\left({\bar{N}}_{12}+2{\hat{N}}_{12}\right)\lambda \mu^{2}+\frac{{\bar{N}}_{11}\lambda }{R_{2}}+\frac{N_{11}\lambda }{R_{1}}=L_{31},\\ \; & L_{14}=N_{13}\lambda^{3}+\left({\bar{N}}_{13}+2{\hat{N}}_{13}\right)\lambda \mu^{2}+\frac{{\bar{N}}_{11}\lambda }{R_{2}}+\frac{N_{11}\lambda }{R_{1}}=L_{41},\\ \; & L_{15}=B_{11}\lambda =L_{51},\;L_{22}=-N_{11}\mu^{2}-{\hat{N}}_{11}\lambda^{2},\\ \; & L_{23}=N_{12}\mu^{3}+\left({\bar{N}}_{12}+2{\hat{N}}_{12}\right)\lambda^{2}\mu +\frac{{\bar{N}}_{11}\mu }{R_{1}}+\frac{N_{11}\mu }{R_{2}}=L_{32},\\ \; & L_{24}=N_{13}\mu^{3}+\left({\bar{N}}_{13}+2{\hat{N}}_{13}\right)\lambda^{2}\mu +\frac{{\bar{N}}_{11}\mu }{R_{1}}+\frac{N_{11}\mu }{R_{2}}=L_{42},\\ \; & L_{25}=B_{11}\mu =L_{52}, \end{array}$$

(34) $$\begin{array}{cc} \; & L_{33}=-J_{1}-\left(\lambda^{2}+\mu^{2}\right)J_{2}-N_{22}\lambda^{4}-\left(\left(2{\bar{N}}_{22}+4{\hat{N}}_{22}\right)\mu^{2}+F_{x}+N_{1}^{0}\right)\lambda^{2}-N_{22}\mu^{4}-\left(F_{y}+N_{1}^{0}\right)\mu^{2}\\ \; & -2\left(\frac{N_{12}\mu^{2}+{\bar{N}}_{12}\lambda^{2}}{R_{2}}\right)-\frac{N_{11}}{R_{2}^{2}}-2\left(\frac{N_{12}R_{2}\lambda^{2}+{\bar{N}}_{12}R_{2}\mu^{2}+{\bar{N}}_{11}}{R_{1}R_{2}}\right)-\frac{N_{11}}{R_{1}^{2}},\\ \; & L_{34}=-J_{1}-\left(\lambda^{2}+\mu^{2}\right)J_{2}-N_{23}\lambda^{4}-\left(\left(2{\bar{N}}_{23}+4{\hat{N}}_{23}\right)\mu^{2}+F_{x}+N_{1}^{0}\right)\lambda^{2}-N_{23}\mu^{4}-\left(F_{y}+N_{1}^{0}\right)\mu^{2}\\ \; & -\frac{\left({\bar{N}}_{12}+{\bar{N}}_{13}\right)\lambda^{2}+\left(N_{12}+N_{13}\right)\mu^{2}}{R_{2}}-\frac{N_{11}}{R_{2}^{2}}-\left(\frac{\left(N_{12}+N_{13}\right)R_{2}\lambda^{2}+\left({\bar{N}}_{12}+{\bar{N}}_{13}\right)R_{2}\mu^{2}+2{\bar{N}}_{11}}{R_{1}R_{2}}\right)\\ \; & -\frac{N_{11}}{R_{1}^{2}}=L_{43},\;L_{35}=-B_{12}\left(\lambda^{2}+\mu^{2}\right)-\frac{B_{11}}{R_{2}}-\frac{B_{11}}{R_{1}}=L_{53},\\ \; & L_{44}=-J_{1}-\left(\lambda^{2}+\mu^{2}\right)J_{2}-N_{33}\lambda^{4}-\left(\left(2{\bar{N}}_{33}+4{\hat{N}}_{33}\right)\mu^{2}+F_{x}+N_{1}^{0}+Q_{44}\right)\lambda^{2}-N_{33}\mu^{4}\\ \; & -\left(F_{y}+N_{1}^{0}+Q_{44}\right)\mu^{2}-2\left(\frac{N_{13}\mu^{2}+{\bar{N}}_{13}\lambda^{2}}{R_{2}}\right)-\frac{N_{11}}{R_{2}^{2}}-2\left(\frac{N_{13}\lambda^{2}R_{2}+{\bar{N}}_{13}\mu^{2}R_{2}+{\bar{N}}_{11}}{R_{1}R_{2}}\right)-\frac{N_{11}}{R_{1}^{2}},\\ \; & L_{45}=\left(-B_{13}+B_{21}\right)\left(\lambda^{2}+\mu^{2}\right)-\frac{B_{11}}{R_{1}}-\frac{B_{11}}{R_{2}}=L_{54},\\ \; & L_{55}=B_{31}\left(\lambda^{2}+\mu^{2}\right)+B_{33}. \end{array}$$

One can easily solve the equation |L|=0 so that the buckling load $\hat{F}$ is obtained.

## 5. Numerical Anaylsis and Discussions

The previously obtained formulations are numerically discussed to explore the impacts of the shallowness ratio, aspect ratio, side-to-thickness ratio, power law index, elastic foundation parameters, graphene weight fraction, and external electric voltage on the critical buckling load of the FG GPLs/piezoelectric nanocomposite doubly curved shallow shells. In addition, several types of doubly curved shallow shells have been considered, such as circular cylindrical shell ($R_{1}/R_{2}=0$), spherical shell ($R_{1}/R_{2}=1$), hyperbolic paraboloidal shell ($R_{1}/R_{2}=-1$), and flat plate ($a/R_{1}=R_{1}/R_{2}=0$). The following data are employed in the current numerical analyses (unless otherwise declared): H=4 mm, $k=s=1,\;n=1,\;a/R_{1}=0.5,\;R_{1}=R_{2},\;\psi_{0}=0.1,\;e_{0}=100,\;a=b,\;{\hat{J}}_{1}=100,\;{\hat{J}}_{2}=10,\;\zeta =1,\;w_{f}=0.1,\;K=10,\;a/H=10,\;a_{G}=2.5\;\mu$m, $b_{G}=1.5\;\mu$m, $h_{G}=1.5\;$nm. Moreover, the following nondimensionalized parameters are employed in the present calculations:

(35) $$\begin{array}{cc} \; & F=\frac{a^{2}\hat{F}}{\pi^{2}D_{G}},\;\;{\hat{J}}_{1}=\frac{a^{4}J_{1}}{D_{G}},\;\;{\hat{J}}_{2}=\frac{a^{2}J_{2}}{D_{G}},\;\;\psi_{0}=\frac{\Psi_{0}}{H^{2}E^{G}},\;\;D_{G}=\frac{H^{3}E^{G}}{12(1-\nu_{G}^{2})}. \end{array}$$

The material properties of the GPLs and polymer piezoelectric matrix are given as [24]

(36) $$\begin{array}{cc} \; & E^{G}=1010\;\mathrm{GPa},\;\rho_{G}=1.06\;{g/\mathrm{cm}}^{3},\;\nu_{G}=0.186,\;E^{m}=1.44\;\mathrm{GPa},\;\nu_{m}=0.29,\\ \; & \rho_{m}=1920\;\;{\mathrm{kg}/m}^{3},\;e_{31}^{m}=e_{32}^{m}=50.535\times 10^{-3}\;{C/m}^{2},\;e_{24}^{m}=e_{15}^{m}=-15.93\times 10^{-3}\;{C/m}^{2},\\ \; & g_{11}^{m}=g_{22}^{m}=0.5385\times 10^{-9}C/\mathrm{Vm},\;g_{33}^{m}=0.59571\times 10^{-9}C/\mathrm{Vm},\;e_{31}^{G}=e_{0}\;e_{31}^{m},\\ \; & e_{24}^{G}=e_{0}\;e_{24}^{m},\;g_{11}^{G}=e_{0}\;g_{11}^{m},\;g_{33}^{G}=e_{0}\;g_{33}^{m}, \end{array}$$

in which $e_{0}$ denotes the piezoelectric multiple.

To validate our analytical formulations, we compare our results with those available in the literature. A comparison of the current critical buckling load *F* of a doubly curved shallow shell under uniaxial and biaxial compressive loads with those obtained by Matsunaga [51] is displayed in Table 1 for different values of the shallowness ratios $a/R_{1}$ and $b/R_{2}$. It is obvious that there is a great agreement between both values of the present critical buckling loads and their analogous values in the results of Matsunaga [51].

Another comparison is prepared in Table 2, where the obtained nondimensional critical buckling load $\left(F^{*}=\hat{F}/HE\right)$ of a doubly curved shallow shell is compared directly with that obtained by Matsunaga [52] employing the classical shell theory (CST), FSDT, and higher-order shell theory (HST). The comparison illustrates that the present results are in excellent agreement with those of [52].

Table 3 reveals the nondimensional critical buckling load *F* of FG GPLs/piezoelectric nanocomposite doubly curved shallow shells under uniaxial and biaxial compressive loads and for various values of the side-to-thickness ratio a/H and shallowness ratio $a/R_{1}$. Various types of FGGPLs (U-GPLs, X-FG, V-FG, and O-FG) are investigated in this table. It is noted that the type U-GPL is stronger than other types; therefore, it needs higher load to buckle. On the other hand, the type O-FG needs the smallest load to buckle. Moreover, the uniaxial and biaxial buckling loads increase as the side-to-thickness ratio a/H increases for all shell types (spherical shell, cylindrical shell, hyperbolic paraboloidal shell, and flat plate). It is also observed that the buckling loads of all FGGPL types of flat plate are the smallest compared with those of both FG spherical and cylindrical shells and also with the hyperbolic paraboloidal shells. This means that the flat plate is weaker than the shells.

The effects of the GPL weight fraction $w_{f}$ and shallowness ratio $a/R_{1}$ on the critical buckling load *F* of various types of FG GPLs/piezoelectric nanocomposite doubly curved shallow shells are depicted in Table 4. It is clear that the uniaxial and biaxial buckling loads increase by increasing the weight fraction. This is because the increase in the GPL components causes an increase in the stiffness of the composite shells. In addition, the critical buckling load of the spherical shells is greater than that of other shell types.

**Figure 3:**
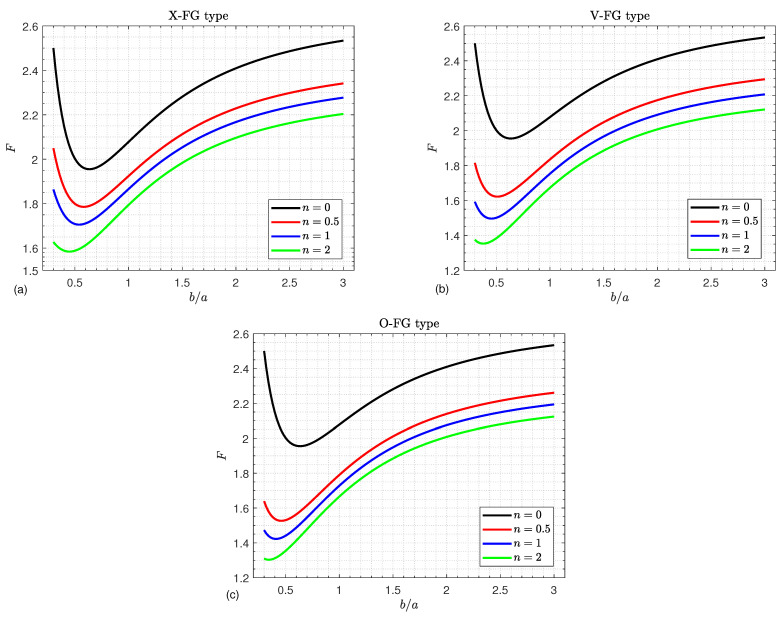
Critical buckling load versus the aspect ratio b/a for various values of the power law index *n* and for (**a**) X-FG, (**b**) V-FG, and (**c**) O-FG types.

Figure 3 illustrates the influence of the power law index *n* and width-to-length ratio b/a on the dimensionless critical buckling load *F* of various types of simply supported FGGPL-reinforced composite doubly curved shallow shells. All obtained results behave in a manner similar to the variation of the aspect ratio and the power law index. They decrease until they reach their minimum and then increase as the aspect ratio b/a increases, whereas they decrease directly as the power law index increases.

**Figure 4:**
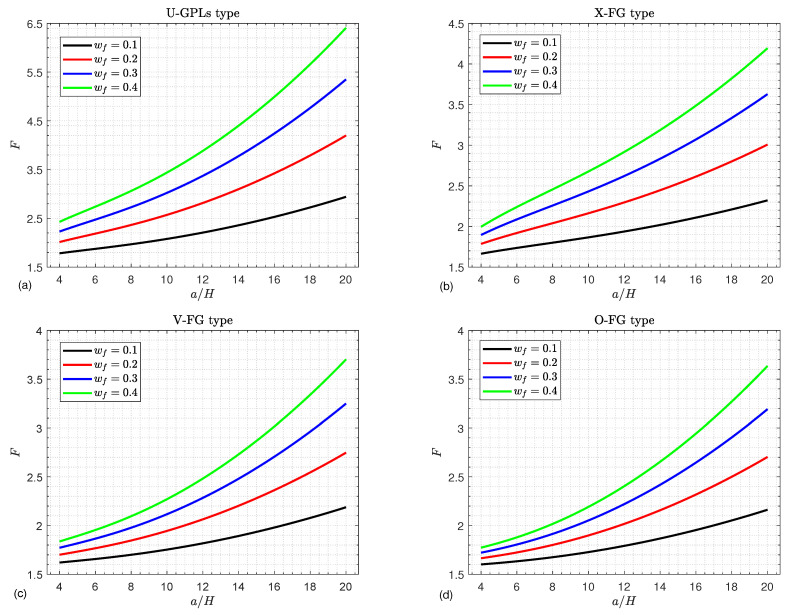
Critical buckling load against the side-to-thickness ratio a/H for various values of the GPL weight fraction $w_{f}$ and for (**a**) U-GPLs, (**b**) X-FG, (**c**) V-FG, and (**d**) O-FG types.

Figure 4 depicts the critical buckling load *F* curves of FGGPL composite doubly curved shallow shells against the side-to-thickness ratio a/H considering various graphene weight fractions $w_{f}$ as well as different graphene distribution types. The graphene components enhance the composite structure stiffness. Consequently, the greater the graphene weight fraction, the greater the buckling load. It is noted that there is a gradual buckling load increase with the increase in the side-to-thickness ratio.

**Figure 5:**
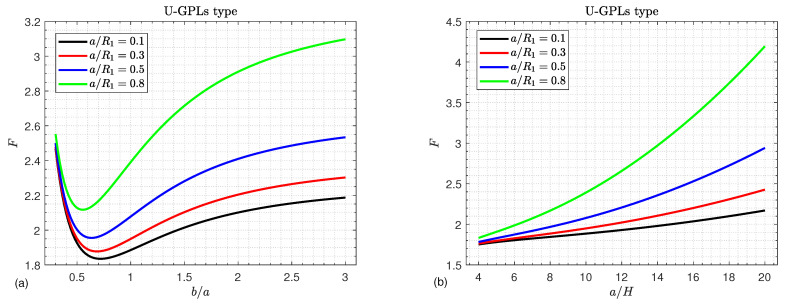
Critical buckling load versus (**a**) the aspect ratio b/a and (**b**) the side-to-thickness ratio a/H for different values of the shallowness ratio $a/R_{1}$.

The effects of the shallowness ratio $a/R_{1}$ on the critical buckling load *F* of U-GPLs/piezoelectric nanocomposite doubly curved shallow shells versus the width-to-length ratio b/a and the side-to-thickness ratio a/H are plotted in Figure 5a,b, respectively. The buckling load *F* increases in a monotonic way with an increase in both the shallowness ratio $a/R_{1}$ and the side-to-thickness ratio a/H, as shown in Figure 5b. Furthermore, the influences of $a/R_{1}$ on *F* are more pronounced for large values of a/H. Furthermore, Figure 5a illustrates that increasing $a/R_{1}$ leads to an increase in the buckling load *F*. However, the buckling load *F* no longer decreases with the increase in the width-to-length ratio b/a. For small values of the shallowness ratio $a/R_{1}$, the buckling load *F* may be independent of the the shallowness ratio $a/R_{1}$. This conclusion is easy to explain because an increase in the $a/R_{1}$ ratio causes the shells to become shallower.

**Figure 6:**
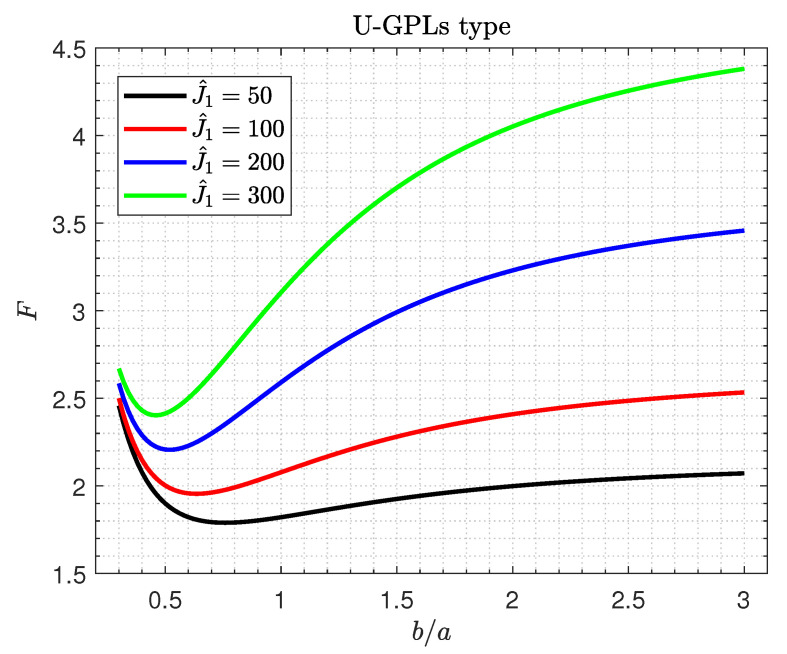
Critical buckling load versus the aspect ratio b/a for various values of Winkler spring stiffness ${\hat{J}}_{1}$.

**Figure 7:**
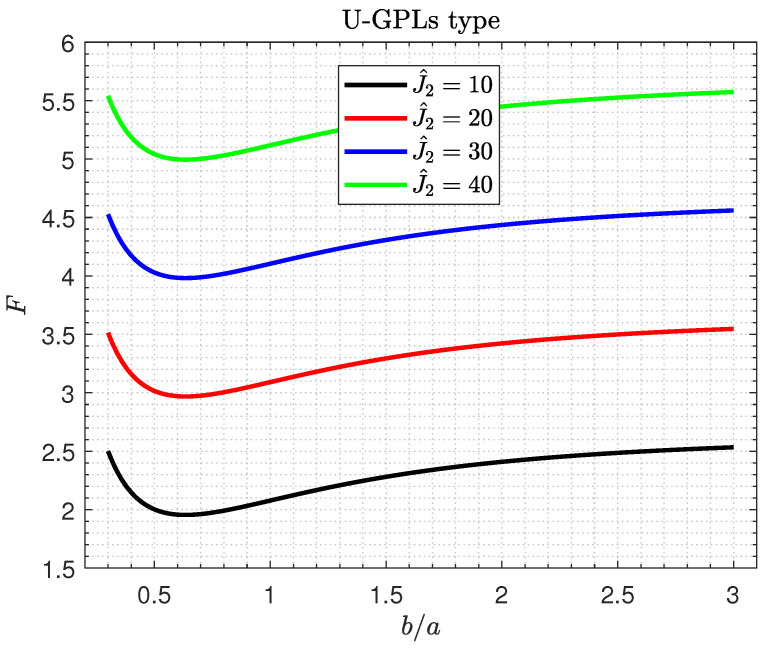
Critical buckling load versus the aspect ratio b/a for different values of the shear foundation stiffness ${\hat{J}}_{2}$.

Figure 6 and Figure 7 display the critical buckling load *F* of GPLs/piezoelectric nanocomposite doubly curved shallow shells against the aspect ratio b/a for various values of Winkler spring stiffness ${\hat{J}}_{1}$ and the shear foundation stiffness ${\hat{J}}_{2}$, respectively.

The results from these figures show that the elastic foundations have positive influences on the critical buckling load of the shells. It is observed that, in Figure 6, the buckling load *F* is affected by the Winkler spring stiffness ${\hat{J}}_{1}$ and the aspect ratio b/a; as they increase, the buckling load *F* increases rapidly. However, the impact of the buckling load *F* may be neglected for small values of b/a. It is also interesting that the maximum values of *F* occur for larger values of the aspect ratio b/a. For all values of the shear foundation stiffness ${\hat{J}}_{2}$, the buckling load increases as the aspect ratio b/a increases.

**Figure 8:**
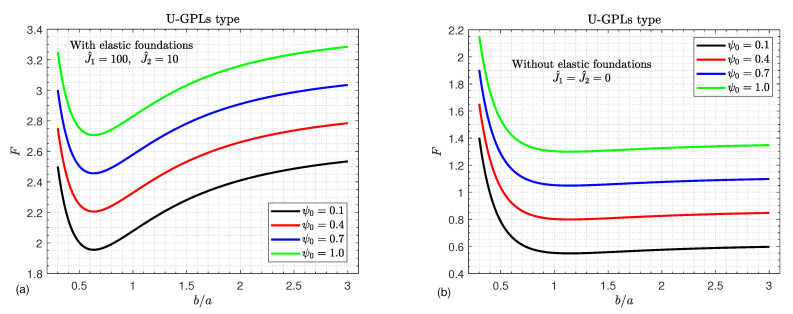
Critical buckling load versus the aspect ratio b/a (**a**) considering the elastic foundations and (**b**) with no elastic foundations, for various values of the external electric voltage $\psi_{0}$.

Figure 8 shows the effects of the aspect ratio b/a and the external electric voltage $\psi_{0}$ on the critical buckling load *F* of GPLs/piezoelectric nanocomposite doubly curved shallow shells. It is evident that with the presence of the elastic foundations, the buckling load *F* decreases until it reaches its minimum value and then increases monotonically as the aspect ratio b/a increases. On the other hand, it is noted that increasing the external electric voltage $\psi_{0}$ enhances the strength of the nanocomposite doubly curved shallow shells; therefore, the buckling load *F* increase directly as the external electric voltage $\psi_{0}$ increases. In contrast to the impact of the external electric voltage $\psi_{0}$ with no elastic foundations, the buckling load *F* suffers a great reduction until it reaches its minimum value and then increases slowly.

## 6. Conclusions

This paper is devoted to investigating the electric potential impacts on the electromechanical buckling of the piezoelectric nanocomposite doubly curved shallow shells reinforced by FGGPLs under simply supported conditions. The refined four-variable shear deformation shell theory is offered with the aim of formulating the displacement field. The graphene platelets are uniformly distributed in each individual layer resting on an elastic foundation and subjected to electric potential and in-plane compressive loads. Furthermore, the stability differential equations are deduced by implementing the principle of virtual work containing both the in-plane compressive load together with the electric load. The accuracy of our present formulations is verified by making some comparisons with rigorous published ones. It is observed that the proposed shell theory is in good agreement with other theories. Furthermore, various numerical examples are presented to illustrate the impacts of several parameters on the critical buckling load. In addition, the influences of the shell geometry, elastic foundation stiffness, GPL volume fraction, and external electric voltage on the mechanical buckling load of the GPLs/piezoelectric nanocomposite doubly curved shallow shells are all illustrated. Upon considering the above parametric studies, we conclude that:

- The GPL weight fraction and GPL distribution types significantly impact the stiffness as well as the dynamical characteristics of structures of the GPLs/piezoelectric nanocomposite doubly curved shallow shells. The GPLs improve high-strength and multifunctional nanocomposite materials. The results emphasize that the U-GPL type has the best mechanical characteristics, while the O-FG type has the weakest stiffness.
- An increase in the elastic stiffness and the aspect ratio b/a leads to an increase in the critical buckling load.
- The sensitivity performance of the critical buckling load of GPLs/piezoelectric nanocomposite doubly curved shallow shells without elastic foundations is reduced by increasing the external electric voltage.
- The critical buckling loads noticeably depend on the dimensions of the shells. They increase as the shallowness ratio and the side-to-thickness ratio increase. Moreover, for small values of the shallowness ratio, the buckling load *F* may be independent of it.
- Increasing the graphene weight fraction enhances the plate stiffness and this leads to a noticeable increase in the critical buckling load.

## Figures and Tables

**Table 1:** Comparison of the nondimensional critical buckling load *F* of a homogeneous doubly curved shallow shell (a/H=10,E=70 GPa, $\nu =0.3,\;a=b,\;{\hat{J}}_{1}={\hat{J}}_{2}=0$).

Load Type	$a/R_{1}$	$b/R_{2}$	Ref. [51]	Present
Uniaxial	0	0	3.7412	3.7866
	0.2	0.2	4.1630	4.2350
	0.2	0	3.8391	3.8987
	0.2	−0.2	3.7100	3.7866
Biaxial	0	0	1.8706	1.8933
	0.2	0.2	2.0815	2.1175
	0.2	0	1.9195	1.9493
	0.2	−0.2	1.8550	1.8933

**Table 2:** Comparison of the nondimensional critical buckling load ($F^{*}=\hat{F}/HE$) of a homogeneous doubly curved shallow shell (ζ=0,E=70 GPa, $\nu =0.3,\;a=b,\;{\hat{J}}_{1}={\hat{J}}_{2}=0$).

a/H	Type	$a/R_{1}$	Ref. [52]			Present
			CST	FSDT	HST	
2	Plate	0	0.64040	0.34790	0.35810	0.37947
	Sph.	0.5	0.64460	0.36270	0.36790	0.40480
		1	0.65560	0.40230	0.39480	0.48079
	Cyl.	0.5	0.61430	0.34440	0.35310	0.38580
		1	0.56850	0.33560	0.34030	0.40480
	Hyp.	0.5	0.56440	0.32200	0.33110	0.37947
5	Plate	0	0.13570	0.11300	0.11400	0.11808
	Sph.	0.5	0.15610	0.13430	0.13410	0.14341
		1	0.21190	0.19270	0.18950	0.21940
	Cyl.	0.5	0.13890	0.11680	0.11740	0.12441
		1	0.14760	0.12710	0.12690	0.14341
	Hyp.	0.5	0.12840	0.10700	0.10790	0.11808
10	Plate	0	0.03557	0.03372	0.03381	0.03423
	Sph.	0.5	0.05921	0.05774	0.05720	0.05956
		1	0.12420	0.12260	0.12160	0.13555
	Cyl.	0.5	0.04104	0.03924	0.03924	0.04056
		1	0.05625	0.05457	0.05435	0.05956
	Hyp.	0.5	0.03381	0.03205	0.03214	0.03423

**Table 3:** Nondimensional critical buckling load *F* of FG GPLs/piezoelectric nanocomposite doubly curved shallow shells for different values of the side-to-thickness ratio a/H and shallowness ratio $a/R_{1}$ (a/b=1.5).

a/H	$a/R_{1}$	$R_{1}/R_{2}$	Uniaxial				Biaxial			
			**U-GPLs**	**X-FG**	**V-FG**	**O-FG**	**U-GPLs**	**X-FG**	**V-FG**	**O-FG**
5	0.5	1	6.4683	6.1167	5.9348	5.8803	1.9903	1.8821	1.8261	1.0691
	0.5	−1	6.2759	6.0204	5.8394	5.7840	1.9311	1.8524	1.7968	1.0516
	0.5	0	6.3507	6.0578	5.8764	5.8215	1.9541	1.8640	1.8081	1.0585
	0	0	6.2425	6.0037	5.8232	5.7673	1.9208	1.8473	1.7918	1.0486
10	0.5	1	10.1233	8.0287	7.7431	7.6875	3.1149	2.4704	2.3825	1.3977
	0.5	−1	6.6440	6.2881	6.0095	5.9469	2.0443	1.9348	1.8491	1.0813
	0.5	0	6.9433	6.4378	6.1582	6.0966	2.1364	1.9809	1.8948	1.1085
	0	0	6.5104	6.2212	5.9437	5.8801	2.0032	1.9142	1.8288	1.0691
20	0.5	1	21.7943	13.9199	13.5845	13.5413	6.7059	4.2831	4.1798	2.4621
	0.5	−1	7.8771	6.9575	6.6422	6.5788	2.4237	2.1408	2.0438	1.1961
	0.5	0	9.0743	7.5564	7.2385	7.1777	2.7921	2.3250	2.2272	1.3050
	0	0	7.3426	6.6901	6.3773	6.3114	2.2593	2.0585	1.9622	1.1475
30	0.5	1	26.9927	16.5649	16.2176	16.1786	8.3054	5.0969	4.9900	2.9416
	0.5	−1	9.9048	8.0162	7.6922	7.6299	3.0476	2.4665	2.3668	1.3873
	0.5	0	12.5985	9.3638	9.0350	8.9775	3.8765	2.8812	2.7800	1.6323
	0	0	8.7023	7.4147	7.0946	7.0283	2.6776	2.2814	2.1829	1.2779
40	0.5	1	36.2940	21.2738	20.9154	20.8847	11.1674	6.5458	6.4355	3.7972
	0.5	−1	12.7400	9.4903	9.1618	9.1012	3.9200	2.9201	2.8190	1.6548
	0.5	0	17.5287	11.8859	11.5502	11.4969	5.3934	3.6572	3.5539	2.0903
	0	0	10.6021	8.4208	8.0981	8.0317	3.2622	2.5910	2.4917	1.4603

**Table 4:** Nondimensional critical buckling load *F* of FG GPLs/piezoelectric nanocomposite doubly curved shallow shells for different values of the GPL weight fraction $w_{f}$ and shallowness ratio $a/R_{1}$ (a/b=1.5).

$w_{f}$	$a/R_{1}$	$R_{1}/R_{2}$	Uniaxial				Biaxial			
			**U-GPLs**	**X-FG**	**V-FG**	**O-FG**	**U-GPLs**	**X-FG**	**V-FG**	**O-FG**
0.1	0.5	1	7.4137	6.6731	6.3923	6.3319	2.2811	2.0533	1.9669	1.9483
	0.5	−1	6.6440	6.2881	6.0095	5.9469	2.0443	1.9348	1.8491	1.8298
	0.5	0	6.9433	6.4378	6.1582	6.0966	2.1364	1.9809	1.8948	1.8759
	0	0	6.5104	6.2212	5.9437	5.8801	2.0032	1.9142	1.8288	1.8093
0.2	0.5	1	9.0369	7.6087	7.0641	6.9572	2.7806	2.3411	2.1736	2.1407
	0.5	−1	7.5612	6.8805	6.3438	6.2290	2.3265	2.1171	1.9519	1.9166
	0.5	0	8.1351	7.1637	6.6233	6.5122	2.5031	2.2042	2.0379	2.0037
	0	0	7.3050	6.7541	6.2212	6.1026	2.2477	2.0782	1.9142	1.8777
0.3	0.5	1	10.5266	8.4577	7.6661	7.5214	3.2389	2.6024	2.3588	2.3143
	0.5	−1	8.3942	7.4157	6.6401	6.4794	2.5828	2.2818	2.0431	1.9937
	0.5	0	9.2235	7.8210	7.0379	6.8846	2.8380	2.4065	2.1655	2.1183
	0	0	8.0240	7.2348	6.4669	6.2985	2.4689	2.2261	1.9898	1.9380
0.4	0.5	1	11.9005	9.2328	8.2095	8.0336	3.6617	2.8409	2.5260	2.4719
	0.5	−1	9.1558	7.9025	6.9049	6.7033	2.8172	2.4315	2.1246	2.0626
	0.5	0	10.2232	8.4198	7.4103	7.2207	3.1456	2.5907	2.2801	2.2217
	0	0	8.6793	7.6715	6.6861	6.4724	2.6706	2.3605	2.0573	1.9915
0.5	0.5	1	13.1732	9.9438	8.7028	8.5011	4.0533	3.0596	2.6778	2.6157
	0.5	−1	9.8561	8.3477	7.1431	6.9050	3.0327	2.5685	2.1979	2.1246
	0.5	0	11.1461	8.9684	7.7470	7.5257	3.4296	2.7595	2.3837	2.3156
	0	0	9.2803	8.0706	6.8832	6.6279	2.8555	2.4833	2.1179	2.0394

## Data Availability

Not applicable.
